# Miniaturized High-Speed FBG Interrogator Based on a Photonic AWG Chip

**DOI:** 10.3390/nano16020089

**Published:** 2026-01-09

**Authors:** Yunjing Jiao, Kun Yao, Qijing Lin, Jiaqi Du, Yueqi Zhao, Kaichen Ye, Bin Sun, Zhuangde Jiang

**Affiliations:** 1 State Key Laboratory for Manufacturing Systems Engineering, Xi’an Jiaotong University, Xi’an 710054, China; 2 School of Mechanical Engineering, Xi’an Jiaotong University, Xi’an 710049, China; 3 School of Instrument Science and Technology, Xi’an Jiaotong University, Xi’an 710049, China; 4 The Higher Educational Key Laboratory for Flexible Manufacturing Equipment Integration of Fujian Province, Xiamen Institute of Technology, Xiamen 361021, China; 5 Xi’an Jiaotong University (Yantai) Research Institute for Intelligent Sensing Technology and System, Xi’an Jiaotong University, Xi’an 710049, China; 6 Collaborative Innovation Center of High-End Manufacturing Equipment, Xi’an Jiaotong University, Xi’an 710049, China; 7 China Mobile (Hangzhou) Information Technology Co., Ltd., Hangzhou 311100, China

**Keywords:** arrayed waveguide grating, FBG interrogator, high-speed sampling response

## Abstract

Although AWGs are widely used in FBG interrogation systems, conventional interrogators are often bulky and hard to deploy, limiting their use in complex field environments. Here, we developed an FBG interrogator based on a photonic AWG chip, comprising a photonic chip module, an optoelectronic detection and processing module, and an output interface module. The AWG chip measures only 280 µm × 150 µm, while the entire interrogator measures just 160 mm × 100 mm × 80 mm, achieving system miniaturization. Wavelength interrogation tests show that the FBG interrogator achieves a wavelength accuracy of 9.87 pm and a high-speed sampling rate of up to 10 kHz, enabling high-precision, real-time FBG demodulation under rapidly varying temperatures. Furthermore, the interrogator was subjected to engineering validation, with dynamic FBG wavelength demodulation experiments conducted under high-temperature shocks in a turbo-engine, verifying its reliability under extreme conditions and demonstrating its potential for broader engineering applications.

## 1. Introduction

Fiber Bragg grating (FBG) sensors, owing to their high sensitivity, resistance to electromagnetic interference, and suitability for multipoint deployment [1,2], have found extensive applications in structural health monitoring, aerospace, and deep-sea exploration [3,4,5,6]. Conventional FBG interrogation approaches typically rely on optical spectrum analyzers or commercial interrogators, which are often bulky and complex, limiting their practical use in field and engineering applications.

At present, commonly used FBG demodulation techniques include edge filtering methods [7,8], matched grating filtering methods [9,10], Mach–Zehnder interferometry [11,12], tunable Fabry–Pérot (F-P) filtering methods [13,14], and CCD-based spectral analysis methods [12,15]. Although these techniques are relatively mature, each exhibits inherent limitations in terms of demodulation accuracy, response speed, and application scenarios. The edge filtering method is applicable to both static and dynamic signal demodulation but is constrained by limited resolution and response speed. The matched grating filtering method features a simple configuration; however, it requires a dedicated matching grating for each sensing FBG, which restricts the multiplexing capacity and results in relatively low demodulation speed. The tunable Fabry–Pérot filtering method offers high demodulation accuracy and excellent filtering performance, but it is unsuitable for dynamic signal measurements. Mach–Zehnder interferometry provides fast response and high resolution for dynamic signal detection; however, its poor immunity to electromagnetic interference makes it unsuitable for long-term monitoring of static wavelength shifts.

With the rapid development of photonic integration technology, on-chip FBG interrogation technology, featuring compactness, low energy consumption, and high performance [16,17], has become a research hotspot. In particular, arrayed waveguide gratings (AWGs) [18,19,20,21,22], with their strong wavelength multiplexing capability, are considered a promising platform for high-performance FBG interrogation [23,24,25,26], and several related studies have been reported [27,28,29,30,31]. For example, Cheben [32] used an AWG-based demodulation system to simultaneously monitor two resonance peaks corresponding to the cladding mode and the Bragg mode. Xu [33] converted the sensing signals of a cascaded FBG-FPI sensor into AWG intensity variations and demodulated the peak wavelengths using a deep learning approach. Su [34] utilized the filtering characteristics of AWGs to capture changes in the FBG central wavelength, thereby improving accuracy and resolution. Yue [35] enhanced the wavelength interrogation performance of AWG-based FBGs using a random forest algorithm. Mateusz [36] further applied AWG chips in medical diagnostics, enabling monitoring of human body temperature and respiration. Marrazzo [37] embedded AWG chips and fabricated circuit boards into an IoT framework, enabling real-time publication of demodulation results on a remote server.

However, most existing work focuses primarily on optimizing chip performance or on related extensions of its applications, while the development of fully integrated, chip-based FBG interrogation systems and their deployment in realistic engineering scenarios remains limited. Considerable efforts have been devoted to expanding the dynamic range of FBG demodulation. In general, the dynamic range can be extended by optimizing the AWG channel spacing and the degree of spectral overlap. For example, Cheben [32] increased the dynamic range by reducing the channel spacing of the AWG. Nevertheless, such approaches are still typically constrained by the 3-dB bandwidth of a single AWG channel and the overlap region between adjacent channels, resulting in a demodulation range that is often discontinuous [38].

To overcome these limitations, Niewczas [39,40] proposed multi-channel power measurement–based demodulation methods to break the 3-dB bandwidth constraint and enlarge the dynamic range. However, these methods require the FBG reflection peak to overlap simultaneously with multiple AWG channel spectra, thereby imposing stricter constraints on the FBG spectral characteristics. Zhuang [41] further introduced a centroid-based demodulation algorithm that exploits the spectral overlap between an FBG and multiple AWG output channels to retrieve the Bragg wavelength, achieving improved demodulation performance.

Different from the above approaches, this work designs the AWG specifically for FBG demodulation applications. By intentionally increasing the output spectral bandwidth of the AWG, larger spectral overlaps are introduced not only between adjacent channels but also between non-adjacent channels, forming multiple continuous usable demodulation regions. During the demodulation process, as the FBG wavelength shifts in response to external physical stimuli, the corresponding demodulation signal can transition smoothly between different AWG channels. As a result, a large dynamic range and continuous wavelength demodulation of a single FBG can be achieved. The detailed structural design and demodulation principle can be found in our previous work [42].

In addition, regarding system-level applications of AWGs, several integrated interrogation schemes have been reported. For instance, Li [43] proposed an on-chip integrated interrogator, but it was only suitable for specific narrowband FBGs and could not operate over a wide temperature range. Similarly, Zhuang [41] developed an on-chip interrogator with enhanced wavelength accuracy, yet system-level integration was not achieved, and performance validation under practical operating conditions was lacking [44].

This study presents a compact, high-speed FBG interrogator based on a photonic AWG chip, achieving full system integration of the photonic chip module, the optoelectronic detection and processing module, and the output interface board module. The AWG chip inside the interrogator measures only 280 µm × 150 µm. The interrogator itself measures only 160 mm × 100 mm × 80 mm, achieves a wavelength accuracy of 9.87 pm, and features a high-speed sampling rate of up to 10 kHz, enabling real-time tracking of FBG wavelength shifts under rapidly changing temperatures. Furthermore, the interrogator was applied in a small turbo-engine subjected to high-temperature shock, successfully performing dynamic FBG wavelength demodulation. The results demonstrate that the system maintains excellent stability and reliability under complex temperature variations and extreme operating conditions, highlighting its potential for broad application in practical engineering environments.

## 2. FBG Interrogator Integration

### 2.1. FBG Interrogation System Design

**Figure 1:**
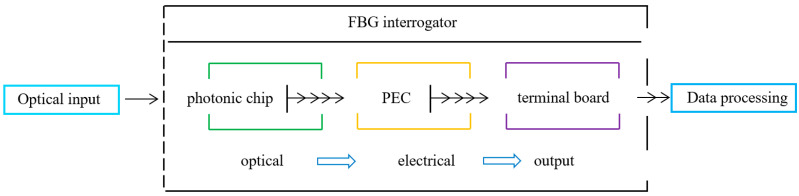
Demodulation system configuration.

This work presents an FBG demodulation system based on a compact interrogator. The overall system architecture is illustrated in Figure 1.

The system consists of three main modules: the photonic chip module, the photoelectric conversion (PEC) module, and the terminal board (TB) module. The overall operation of the interrogator is as follows: first, the photonic chip module receives the FBG reflection spectra via an external optical interface and performs wavelength demodulation within the AWG, after which the optical signals from each channel are transmitted to the PEC module; next, the PEC module converts each optical signal into high-precision, low-noise voltage signals; then, all voltage signals are aggregated through the output interface board and transmitted to an external data acquisition system; finally, the data acquisition system digitizes the input signals to obtain the FBG wavelength demodulation results and the corresponding measured physical quantities.

### 2.2. Photonic Chip Module

**Figure 2:**
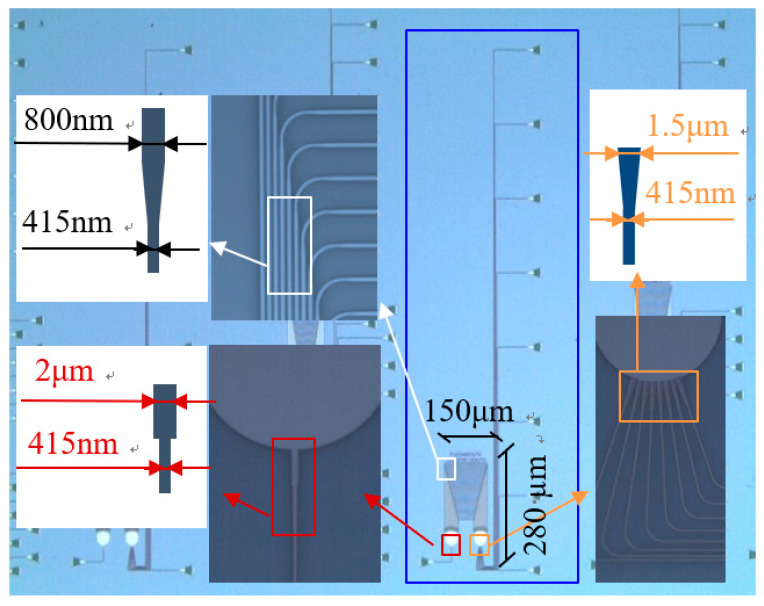
Structure of the 1 × 8 AWG.

The photonic chip inside the interrogator is based on an SOI substrate, featuring a 220 nm top silicon layer and a 3 μm buried oxide layer. The core structure of the photonic chip is a 1 × 8 AWG. Figure 2 shows a microscopic image. The core area of the AWG is 300 × 150 μm^2^, and the overall footprint, including all input and output waveguides, is approximately 0.5 × 1.8 mm^2^. A taper and MMI are inserted between the first star coupler and the input waveguide to broaden the output spectral bandwidth. A shallow-etched taper is connected between the second star coupler and the output waveguides to reduce mode-conversion loss. To further reduce the sensitivity of the waveguides to fabrication-induced variations (e.g., width fluctuations) in effective refractive index, each waveguide in the array waveguides of a wide straight waveguide of 800 nm, a narrow single-mode waveguide of 415 nm, and a 2 μm-long tapered, providing a smooth transition between wide and narrow waveguides and enhancing fabrication tolerance. Table 1 presents the design parameters.

**Figure 3:**
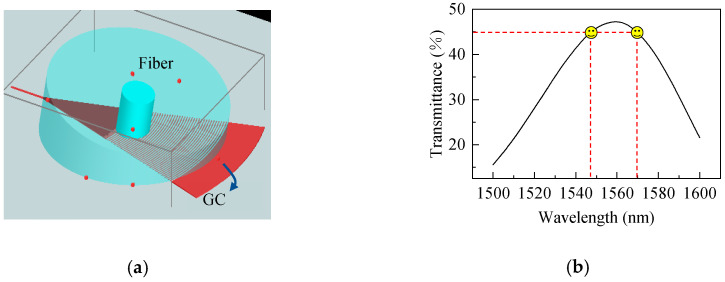
Fiber-to-chip coupling using a grating coupler. (**a**) Schematic of fiber coupling with a grating coupler; (**b**) Simulated transmittance of the grating coupler.

To achieve efficient fiber-to-chip coupling, vertical grating couplers (GCs) was introduced at the input and output ports of the AWG, as shown in Figure 3a. The coupling efficiency between the fiber and the GC was simulated and optimized using a finite-difference time-domain (FDTD) method, resulting in the following design parameters: a grating period of 670 nm, an etch depth of 100 nm, a duty cycle of 0.4, 25 grating periods, and a fiber incidence angle of 8°. Simulation results indicate that over 45% of the optical power is successfully coupled into the chip within the wavelength range of 1547–1570 nm (Figure 3b). Compared with the typical upper limit of uniform GC coupling efficiency of approximately 50% [45], the coupling efficiency achieved in this design approaches the theoretical maximum while fully meeting the chip’s optical power requirements, ensuring that the AWG operates with sufficient optical power for reliable wavelength demodulation.

To fully integrate the AWG photonic chip into the interrogator and achieve high-precision, long-term stable demodulation of FBGs in practical applications, the fabricated chip was optically packaged.

First, in terms of optical coupling, vertical fiber-to-chip coupling was implemented using the vertical grating couplers fabricated on the chip. The grating couplers (GCs) at the AWG outputs were arranged with a 250 μm pitch, and a custom 8-channel SMF-28 fiber array was designed to match this configuration. Each fiber end was equipped with a miniature spherical lens to further enhance the coupling efficiency and increase tolerance to slight angular misalignments. Precise alignment between the fiber array and the photonic chip was achieved using a high-precision six-degree-of-freedom displacement platform, enabling stable and efficient optical coupling.

**Figure 4:**
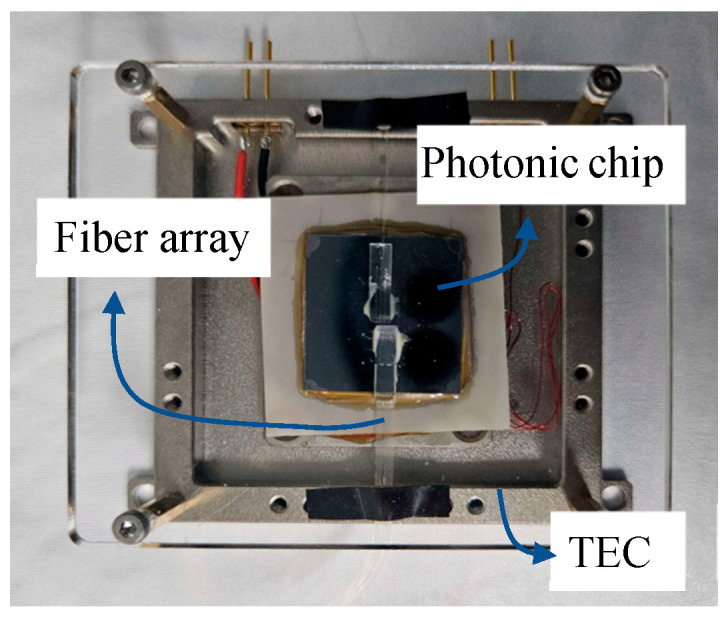
Packaged photonic chip module.

Second, regarding the packaging structure and environmental stability, a silicon-dioxide protective layer was deposited on the chip surface to mitigate the impact of trace water vapor, dust, and chemical contaminants on the on-chip optical paths. Accordingly, a non-hermetic enclosure was adopted, allowing free exchange of gases and moisture between the inside and outside of the cavity, which helps alleviate internal pressure fluctuations and local heat accumulation. In addition, a TEC unit was installed at the bottom of the enclosure to maintain the chip at a stable operating temperature of 25 °C, thereby effectively suppressing drift of the AWG output spectrum. The fully packaged photonic chip module is shown in Figure 4.

**Figure 5:**
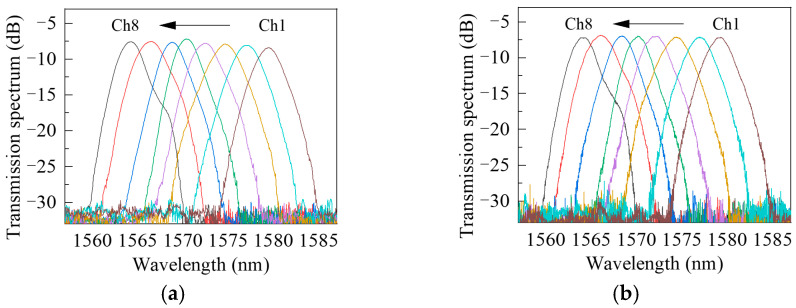
AWG output spectra before and after packaging: (**a**) Before packaging; (**b**) After packaging.

Spectral measurements were performed on the packaged photonic chip (as shown in Figure 5) to verify the reliability of the optical packaging. During laboratory testing, the photonic chip was tested on a six-degree-of-freedom positioning platform, where the fiber and chip were precisely aligned but not permanently fixed; the corresponding pre-packaging spectra are shown in Figure 5a. After packaging, the fiber and photonic chip were securely fixed, and the resulting spectra are shown in Figure 5b. The results show that the noise level of the characteristic spectra in all channels remains below −30 dB after packaging. The insertion loss of the central channel is −7.11 dB (Figure 5b), which is essentially unchanged compared with the pre-packaging value of −7.78 dB (Figure 5a). In addition, the center-wavelength shift in each characteristic spectrum is less than 0.01 nm, and the spectral bandwidth variation is below 1%, both within the acceptable error range. For the packaged AWG, the 3-dB bandwidth of each channel is 2.8 nm, and the wavelength spacing between channels is 2.1 nm. Comparison of the spectra before and after packaging confirms that the packaging process does not cause significant performance degradation. These findings indicate that the optical packaging effectively preserves the consistency of the output spectra, providing a stable optical foundation for the subsequent integration of the interrogator modules.

### 2.3. Photoelectric Conversion Module

**Figure 6:**
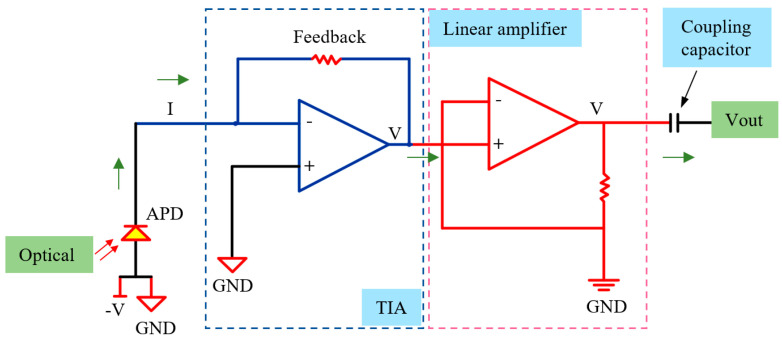
Optical-to-electrical conversion chain.

Considering that the optical power output from the photonic chip is only −45 dBm to −60 dBm, a photodetection module based on a diode was designed and implemented in this work, comprising an avalanche photodiode (APD), a transimpedance amplifier (TIA), a linear amplifier, and a coupling capacitor. The overall signal processing flow is illustrated in Figure 6.

The detailed design of the module is as follows. First, the incident optical signal is converted into a photocurrent. A photodiode was chosen as the detector, which is a type of semiconductor device capable of converting incident optical signals into current. Common types include PIN photodiodes (PIN PDs) and APDs. A conventional PIN PD, however, does not possess an intrinsic current amplification mechanism; its output current is strictly determined by the incident optical power. In contrast, an APD also converts photons into photocurrent through photo-generated carriers but features an internal avalanche multiplication mechanism, which can amplify the photocurrent by tens to hundreds of times, thereby significantly enhancing the detection of weak optical signals. To enhance the detection sensitivity for weak optical signals, an avalanche photodiode was therefore selected. The APD has a minimum detectable optical power of −70 dBm and a saturation power of up to 10 mW, fully covering the detection range of this work. Considering the overall system sensitivity, signal-to-noise ratio, and long-term stability, an APD with a multiplication factor of 26 and a responsivity of 0.9 A/W was employed to convert the incident optical signal into a photocurrent. The output current of an APD can be expressed as follows:

(1) $$I_{\mathrm{APD}}=P\cdot R\cdot M$$

where *P* is the output optical power, *R* is the responsivity (A/W), *M* denotes the multiplication factor, i.e., the internal gain of the APD that amplifies the photogenerated current.

Although the internal multiplication mechanism of the APD amplifies the photocurrent, its output remains at the nanoampere level. According to Equation (1), the resulting output current ranges from 23.4 nA to 739 nA. Directly feeding it into a conventional voltage amplifier would not only result in insufficient amplitude but also make the signal susceptible to thermal and shot noise. Therefore, a transimpedance amplifier was used for the first-stage conversion from photocurrent to voltage. The TIA has a 3-dB bandwidth of 30 MHz and a feedback resistance of 1.77 kΩ. After this TIA-based conversion and amplification, the output voltage ranges from 41.4 µV to 1.31 mV.

Since the voltage output from the TIA is still relatively low to meet the requirements of the subsequent data acquisition, a linear amplifier was introduced for secondary voltage amplification. The linear amplifier was implemented using an operational amplifier (Op-Amp), which can amplify weak voltage signals while maintaining a linear relationship between input and output. The amplifier was powered with 5 V, and the bias voltage was set at 2.5 V to fully utilize the supply range and ensure that the output signal remains within the amplifier’s linear operating region. The gain was carefully chosen to balance signal linearity and amplitude limitations: excessive gain could cause the weak signal to approach the supply or bias limits, resulting in nonlinearity or saturation. After consideration, a gain of 50 was selected. As a result, the output voltage after amplification ranges from 2.50207 V to 2.5655 V, with 2.5 V representing the bias voltage.

Since the output of the linear amplifier contains a 2.5 V bias voltage, directly feeding this signal into the data acquisition module would consume part of its voltage range, reducing resolution and ultimately affecting the FBG demodulation accuracy. Therefore, a coupling capacitor was added at the output to block the bias voltage while fully transmitting the voltage signal that varies with the FBG wavelength, thereby ensuring the linearity and accuracy of subsequent signal acquisition and demodulation. After passing through the coupling capacitor, the output voltage signal ranges from 2.07 mV to 65.5 mV.

The resulting photodetection and electronic conversion module achieves a total transimpedance gain of 2.1 × 10^6^ V/W, converting optical powers ranging from −45 dBm to −60 dBm into alternating current voltage signals of approximately 2.07 mV to 65.5 mV, thereby meeting the amplitude requirements for spectral demodulation.

### 2.4. Terminal Board and Standardized Interface Design

To centralize the output voltage signals and facilitate their processing by the data acquisition system, a terminal board (TB) was implemented. All output channels can be routed to the TB, providing a safe, stable, and unified voltage output, and ensuring reliable input paths for subsequent data acquisition devices.

Regarding interface selection for each module, the external optical signal is connected to the photonic chip input via an FC/APC fiber interface. Inside the interrogator, the output of the photonic chip is transmitted to the PEC module using the same FC/APC interface. The output of the PEC module features an SMA connector and can be connected to the TB using a set of jumper wires, with one end terminated by the SMA connector and the other end consisting of tin-plated copper leads directly soldered to the TB. Finally, the TB interfaces with the data acquisition system through a DB25 connector, ensuring reliable and compatible signal transmission throughout the system.

### 2.5. Interrogator Integration

**Figure 7:**
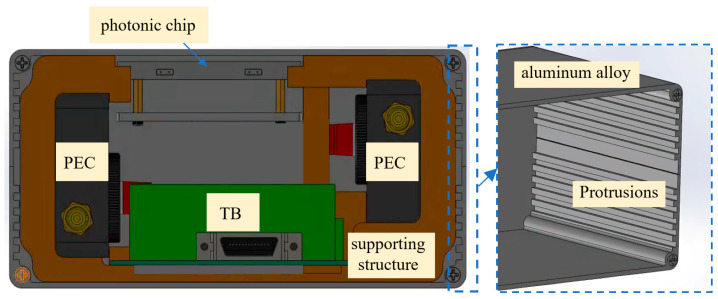
Layout design of the Interrogator.

The layout design adopts a vertically stacked arrangement of the functional modules, as shown in Figure 7. The photonic chip module, which requires direct connection to optical fibers, is positioned at the top of the assembly and securely fixed to minimize interference from other modules. Sufficient space is reserved for fiber routing to ensure smooth optical transmission.

The optoelectronic detection and front-end processing modules handle the eight optical output channels through two collaborative units. These modules are placed on either side of the photonic chip, facilitating fiber splitting and convenient connection to the respective detection modules.

The terminal board is located at the bottom of the assembly, directly beneath the photonic chip, with enough clearance to allow reliable connections between the optoelectronic modules and the output interface board. This arrangement also provides structural support to maintain overall stability.

**Figure 8:**
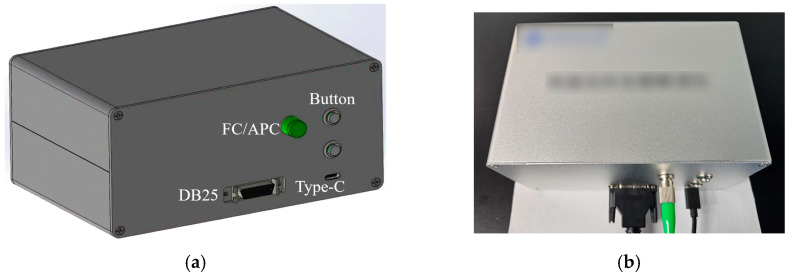
Appearance of the interrogator. (**a**) interface layout; (**b**) photograph of the device.

The enclosure of the interrogator is made of aluminum alloy, consisting of four panels in a design that splits the top and bottom halves and closes from front to back. Protrusions on the inner sidewalls prevent the supporting structure from shifting, ensuring the modules remain securely fixed. Gaps between the panels are maintained to optimize heat dissipation, as illustrated in Figure 7. The front panel houses standard interfaces, including an FC/APC port for coupling with the photonic chip input, a DB25 connector for the output interface board, control switches for the optoelectronic modules, and a Type-C power port, as shown in Figure 8a.

Once the supporting structure is mounted inside the aluminum enclosure, screws are used to secure all components, ensuring overall structural stability and reliability. The final design results in an interrogator with dimensions of 160 mm × 100 mm × 80 mm, and the overall appearance is shown in Figure 8b.

## 3. System Characterization and Validation Tests

### 3.1. Evaluation of Wavelength Accuracy

**Figure 9:**
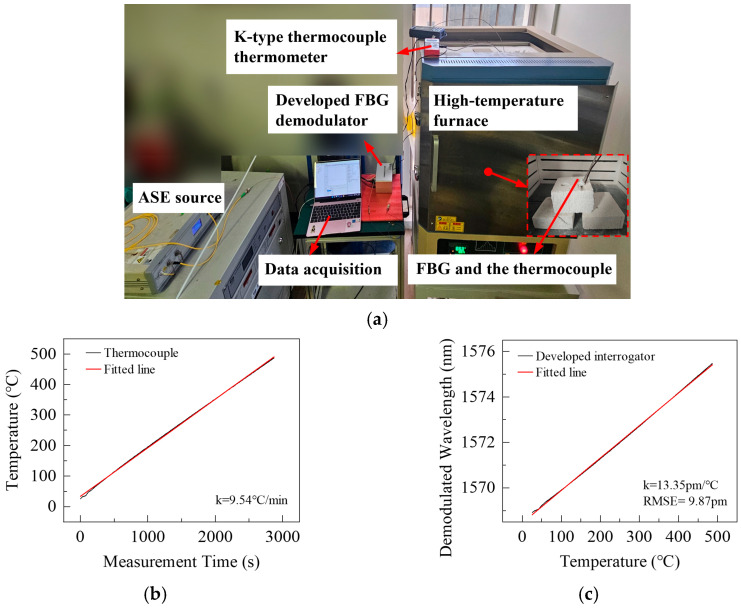
Wavelength demodulation accuracy test. (**a**) High-temperature furnace setup; (**b**) Thermocouple-measured heating profile; (**c**) Temperature–demodulated wavelength relationship.

For evaluating the wavelength demodulation accuracy of the developed interrogator, a high-temperature furnace heating experiment was conducted, with the experimental setup shown in Figure 9a. The optical input was provided by an ASE broadband source, while the tested FBG and a thermocouple were embedded in refractory bricks to minimize fiber drift and ensure uniform temperature in the region containing both the thermocouple and the FBG. The FBG was connected to the interrogator through a top vent, and a host computer was used for data acquisition and wavelength demodulation. The tested FBG was fabricated by femtosecond laser inscription, with a central wavelength of 1569.02 nm, a 3-dB bandwidth of 0.28 nm, and a reflectivity of 90%. The furnace heating program was set to a ramp rate of 10 °C/min up to a maximum temperature of 500 °C.

Figure 9b shows that the temperature measured by the thermocouple increases linearly, with minor fluctuations at the beginning. The fitted heating rate was approximately 9.54 °C/min, consistent with the programmed value, ensuring a linear wavelength drift of the tested FBG. The maximum monitored temperature reached about 487 °C, slightly lower than the program setting due to temperature nonuniformity inside the furnace. However, since this experiment focuses on the continuous temperature variation process, and the thermocouple and the FBG were fixed at the same position for temperature calibration, the temperature measured by the thermocouple was regarded as the actual temperature for subsequent analysis, while the programmed temperature setpoint was not used as a reference. Figure 9c shows the correspondence between temperature and demodulated wavelength. The demodulated wavelength was calculated using the relative optical intensity method, and the specific algorithmic details can be found in our previous work [46]. The total wavelength shift was approximately 6.5 nm, corresponding to a temperature sensitivity of about 13.35 pm/°C. Based on the relative intensity demodulation algorithm, the experimental data were linearly fitted, and the root mean square error (RMSE) of the fit represents the wavelength demodulation accuracy. The RMSE was 9.87 pm, indicating that the developed FBG interrogator can accurately track temperature variations with a wavelength demodulation accuracy of 9.87 pm.

### 3.2. Evaluation of Dynamic Response

**Figure 10:**
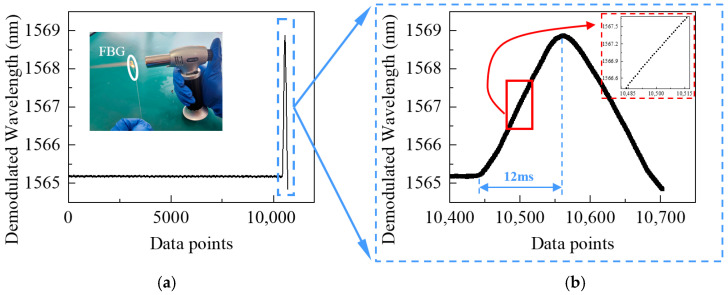
FBG dynamic response under flash heating. (**a**) Wavelength interrogation results; (**b**) Amplified results.

For evaluating the dynamic response of the developed interrogator, a flash-heating test using a blowtorch was designed. The experiment was conducted at room temperature (Figure 10a), where the FBG was subjected to rapid heating by the blowtorch, and the interrogator was operated at a sampling rate of 10 kHz. The FBG, fabricated via femtosecond laser inscription, had a central wavelength of 1565.19 nm, a 3-dB bandwidth of 0.31 nm, and a reflectivity of 70%.

The results show that the FBG’s initial wavelength remained stable at 1565.19 nm under room temperature. At approximately 1 s, the FBG experienced a sudden temperature increase, and the interrogated wavelength rapidly rose to 1568.85 nm within 12 ms, which corresponds to the transient response of the FBG itself, captured accurately by the interrogator. Considering the FBG’s 13.35 pm/°C sensitivity, the wavelength shift implies a temperature increase near 274 °C, with a corresponding heating rate of 22,846 °C/s. During this process, the interrogator collected around 120 data points, and the amplified wavelength trace (Figure 10b) shows a uniform distribution and clear trend, with no signal loss or noticeable lag.

The experimental result confirms that the developed interrogator can provide stable and continuous wavelength measurements under extreme heating conditions. Its output wavelength tracks temperature variations in real time at a sampling rate of 10 kHz—twice that of conventional spectrally scanning interrogators (e.g., MOI-si155)—fully demonstrating its capability to capture rapid FBG wavelength changes and indicating excellent dynamic response performance.

### 3.3. Validation of FBG Demodulation Under High-Temperature Shock

**Figure 11:**
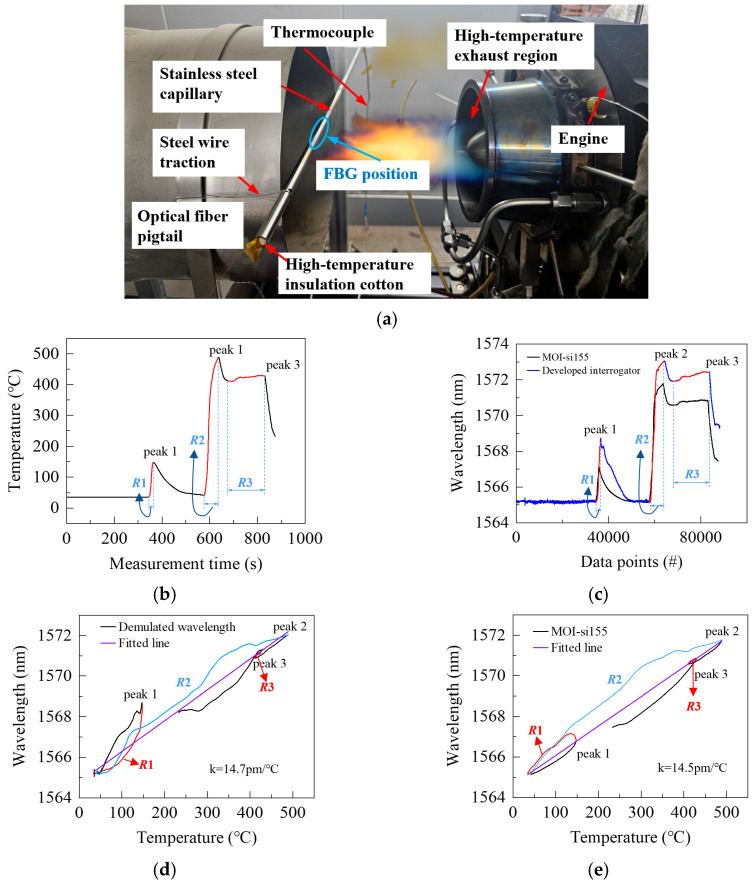
Interrogation results under high-temperature conditions. (**a**) Experimental Setup of FBGs on a small turbo-engine; (**b**) Temperature measured by the thermocouple; (**c**) Comparison of wavelength interrogation results from the two interrogators; (**d**,**e**) Temperature–wavelength responses of the two interrogators.

For evaluation of the developed interrogator under practical conditions, two FBGs and a thermocouple were deployed at the exhaust nozzle of a small turbo-engine to undergo high-temperature shock tests. They were integrated into a stainless-steel capillary tube, which was horizontally mounted at the engine nozzle using a stainless-steel wire. The developed interrogator monitored one FBG, while a commercial system (MOI-si155) simultaneously monitored the other, with the thermocouple providing reference temperature. The tube was sealed at both ends with high-temperature insulation cotton, and the sensing region was marked in red to ensure precise placement within the hot gas stream. After the test, the red mark was completely burned off, confirming exposure to extreme thermal conditions (Figure 11a). The two FBGs, fabricated via femtosecond laser inscription in single-mode fibers, had central wavelengths of 1565.21 nm and 1565.20 nm, with 3-dB bandwidths of 0.29 nm and 0.32 nm, and reflectivity of 65% and 60%, respectively.

The small turbo-engine was operated under varying power and load conditions, undergoing three heating cycles that produced three temperature peaks. Figure 11b illustrates the temperature variations recorded by the thermocouple, clearly showing the process and the three heating stages, labeled R1, R2, and R3.

The wavelength interrogation results obtained from the two interrogators are presented in Figure 11c, where three distinct temperature peaks are also observed, in close agreement with the thermocouple measurements (Figure 11b). The developed interrogator exhibited interrogation trends highly consistent with those of the commercial system (MOI-si155), with only minor differences in wavelength shift amplitude. These discrepancies mainly arose from the slightly different temperature–wavelength responses of the two FBGs. Nevertheless, the results demonstrate that the developed interrogator is capable of real-time tracking of complex dynamic temperature variations.

The comparison between wavelength shift and temperature (Figure 11d,e) indicates that the temperature–wavelength coefficient of the developed interrogator is approximately 15.2 pm/°C, while that of the commercial system is around 14.5 pm/°C. Although both interrogators exhibit some deviations from perfect linearity, they still reliably capture the overall temperature trends. These deviations are mainly attributed to the effects of stress, micro-bending, or additional strain on the FBGs under varying environmental conditions, which can slightly alter their temperature–wavelength response. Nevertheless, the three heating cycles and their corresponding temperature peaks remain clearly distinguishable, demonstrating that the developed interrogator maintains good dynamic resolution and reliability in complex field environments. The results confirm that the system can perform rapid, continuous, and stable temperature measurements under high-temperature shocks and demanding operating conditions, providing a promising approach for high-temperature dynamic monitoring in applications such as aero-engines or explosive testing scenarios.

## 4. Discussion and Conclusions

We developed a compact, photonic-chip-based FBG interrogator that achieves system-level integration compared with previous works [47], and its performance was validated under realistic conditions by deploying it with a small turbo-engine [44]. The interrogator measures only 160 × 100 × 80 mm, achieving a miniaturized design that is significantly more portable and lightweight compared to conventional spectrometers and FBG interrogators. The system demonstrates a high static wavelength accuracy of up to 9.87 pm, showing competitive performance among previously reported photonic-chip-based FBG interrogators, while its sampling rate reaches 10 kHz—approximately twice the speed of commercial spectral-scanning interrogators such as the MOI—allowing real-time tracking of rapid temperature variations. Even under ultrafast temperature changes as high as 22,846 °C/s, the system is capable of achieving precise and real-time wavelength demodulation. Performance comparisons with other photonic integrated commercial interrogators show that its dynamic response is equally competitive (see Table 2). Furthermore, this study represents the first demonstration of the interrogator’s performance in an actual application scenario. The results confirm that it maintains reliable and stable operation under complex temperature changes and extreme environments, providing strong support for extending FBG sensing to a wider range of engineering applications.

Nevertheless, the current system has some limitations. Its level of integration remains limited, requiring an external broadband source and a data acquisition unit, which increases deployment complexity. The interrogation accuracy is constrained by the discrepancy between the AWG output spectrum and the ideal Gaussian profile, as the demodulation algorithm performs best when the spectral shape is ideally matched. Future work will focus on integrating the light source and photodetector modules on-chip to realize a fully integrated FBG interrogator, enabling broader applicability in demanding environments such as aerospace engines and high-speed thermal monitoring.

## Figures and Tables

**Table 1:** Parameters of 1 × 8 AWG.

Category	Value
Center wavelength (nm)	1570
Diffraction order	22
Arrayed waveguide path length difference (μm)	14
Number of arrayed waveguide channels	35
Channel spacing (nm)	2
Free spectral range (nm)	41
Diameter of the Rowland circle (μm)	45

**Table 2:** Comparison between proposed chip and other works.

Category	Wavelength Accuracy (pm)	Dynamic Response (kHz)	Size (mm^3^)	Methods
FBGT-100	5	2	18.5 × 18.5 × 50	AWG
MOI-Si155	1	5	206 × 274× 9	Tunable FP Filter
FBGA-S	1	5	130 × 73 × 15.7	Spectrometer
GTR	5	19.2	110 × 130 × 47	AWG
This work	10	10	160 × 100 × 80	AWG

## Data Availability

The data presented in this study are available on request from the corresponding author. The data are not publicly available due to privacy.
